# How People Living With Amyotrophic Lateral Sclerosis Use Personalized Automatic Speech Recognition Technology to Support Communication

**DOI:** 10.1044/2024_JSLHR-24-00097

**Published:** 2024-07-11

**Authors:** Richard Cave

**Affiliations:** a University College London, United Kingdom

## Abstract

**Purpose:**

Amyotrophic lateral sclerosis (ALS) is a progressive, ultimately fatal disease causing progressive muscular weakness. Most people living with ALS (plwALS) experience dysarthria, eventually becoming unable to communicate using natural speech. Many wish to use speech for as long as possible. Personalized automated speech recognition (ASR) model technology, such as Google's Project Relate, is argued to better recognize speech with dysarthria, supporting maintenance of understanding through real-time captioning. The objectives of this study are how plwALS and communication partners use Relate in everyday conversation over a period of up to 12 months and how it may change with any decline in speech over time.

**Method:**

This study videoed interactions between three plwALS and communication partners. We assessed ASR caption accuracy and how well they preserved meaning. Conversation analysis was used to identify participants' own organizational practices in the accomplishment of interaction. Thematic analysis was used to understand better the participants' experiences of using ASR captions.

**Results:**

All plwALS reported lower-than-expected ASR accuracy when used in conversation and felt ASR captioning was only useful in certain contexts. All participants liked the concept of live captioning and were hopeful that future improvements to ASR accuracy may support their communication in everyday life.

**Conclusions:**

Training is needed on best practices for customization and practical use of ASR technology and for the limitations of ASR in conversational settings. Support is needed for those less confident with technology and to reduce misplaced allocation of ownership of captioning errors, risking negative effects on psychological well-being.

Amyotrophic lateral sclerosis (ALS), also known as motor neurone disease (MND) or Lou Gehrig's disease, is a progressive and ultimately fatal disease causing progressive muscular weakness resulting in loss of function of the limbs, trunk, and neck. There is no cure, and the average life expectancy from symptom onset is commonly 2–3 years, with approximately 25% of people surviving for 5 years and 10% surviving for 10 years (Oliver et al., 2017).

Approximately one third of people living with ALS (plwALS) experience dysarthria as an initial symptom (Yorkston, 2007), and most experience dysarthria sometime during the course of the condition (Ball et al., 2004b). As a result, more than 80% of plwALS will become unable to communicate their daily needs using natural speech (Beukelman et al., 2011), and in time, most will be unable to speak at all (Beukelman, Garrett, & Yorkston, 2007), frequently within 18 months from first symptoms (Makkonen, Ruottinen, Puhto, et al., 2018). Up to 90% of plwALS eventually rely on augmentative and alternative communication (AAC) to support daily communication (Ball et al., 2004a).

## Speech and Identity

PlwALS often consider the likely loss of speech as one of the worst aspects of ALS (Hecht et al., 2002). Speech is a powerful medium of identity (Bucholtz & Hall, 2005). It communicates mood, humor, geographical, social and educational background, health status, and gender—as well as the content of the message (Nathanson, 2017). Although most plwALS eventually rely on AAC to support daily communication (Ball et al., 2004a), for many, there is nothing that could replace the ease or speed of natural speech, citing the extended time it takes to spell a message with AAC (Murphy, 2004). Using speech to communicate may remain the first preference in most situations (Smith & Connolly, 2008), even if they are often not easily understood (Beukelman, Fager, et al., 2007).

A voice output communication aid (VOCA; a type of AAC) is often provided to plwALS when it becomes difficult to communicate using speech (Makkonen, Ruottinen, Korpijaakko-Huuhka, & Palmio, 2018). A VOCA is operated by choosing letters, words, symbols, or sentences in the most accessible way for the person (which could be using fingers, a keyboard, a pointer, an adapted mouse, a joystick, a switch, or eye tracking). The VOCA then “speaks” the message using a synthetic representation of a generic human voice. VOCAs are argued to improve increased quality of life by promoting autonomy, allowing for greater participation in the decision-making process, improving self-esteem, and helping to support relationships (Brownlee & Palovcak, 2007).

Identity maintenance and relationship management can be impaired by the limitations of AAC devices as well as the disease (Kane et al., 2017), partly because communication using an AAC device such as a VOCA is frequently slow, around 8–20 words per minute, and, as a result, not well equipped to facilitate talk during verbal conversation due to the rapid timing demands (Seale et al., 2020). People may use vocalizations and gesture to try to speed up communication (Seale et al., 2020) and choose not to communicate beyond their basic needs (Waller, 2019).

Although most plwALS eventually rely on AAC to support daily communication (Ball et al., 2004a), for many, there is nothing that could replace the ease, accuracy, and speed of natural speech, citing the extended time it takes to spell a message with AAC (Murphy, 2004). The slowness of using AAC to communicate can be a barrier to AAC adoption (Peters et al., 2015). PlwALS may attempt to speed up communication by typing short phrases, forming sentences that are not syntactically complete, or using preprogrammed messages. However, by doing this, their communication may be less accurate, or the output may not fully overlap with their communication intent (Fried-Oken et al., 2023). Therefore, enhancement of speed may come at the expense of accuracy, or vice versa, and this trade-off may feel particularly important to plwALS as they begin to experience progressive communication challenges (Fried-Oken et al., 2023).

Voice banking is technology that may indicate the importance of natural speech as a part of personal identity for plwALS. Voice banking is a process for creating a “personalized synthetic voice” (PSV), a synthetic approximation of a person's natural voice (Costello, 2016). Voice banking is becoming increasingly popular for plwALS, possibly because many may perceive a PSV could help to maintain a sense of personal identity by preserving the way that they speak—as opposed to what a voice bank really does, that is, preserve elements of the way their voice sounds when using AAC (Cave & Bloch, 2021). PlwALS who voice bank may not appreciate that a PSV is unlikely to change the speed of communication using AAC (Cave & Bloch, 2021).

Automated speech recognition (ASR) technology is the process where a device such as a smartphone or computer converts a speech signal into words in text (Young & Mihailidis, 2010). ASR technology has multiple uses: It can convert speech to text, find information online, enable hands-free computing such as dictating e-mails, and many other uses. It can help with environmental control, such as changing the channel on a television as well as adjusting lighting and heating (Laricchia, 2020).

ASR model performance is frequently evaluated using word error rate (WER; Mishra et al., 2011). WER is a measure of the number of full-word matches between the hypothesized model transcript and the ground-truth human transcript. It is the summation of substituted, deleted, and inserted words divided by the total number of words in the ground-truth transcript. ASR word accuracy for commercial systems can be as high as 95% for many speakers with unimpaired speech (Synnaeve et al., 2020) and improving due to increased computational power of deep learning systems and large training data sets (MacDonald et al., 2021).

However, the challenge of accurate ASR recognition of speech with dysarthria may be a difficult problem to solve, as articulation, tempo, rhythm, and volume are frequently highly variable (Moore et al., 2018). The speech of plwALS may be particularly challenging due to progressive dysarthria, the changing strategies plwALS may use to be understood (Caballero-Morales & Trujillo-Romero, 2014), and fatigue, adding to speech variability (Sanders et al., 2002).

The more normalized voice interaction becomes, the more that individuals living with impaired speech risk become excluded from current, commercially available technologies—even though they could benefit extensively (Koch Fager et al., 2019). Voice interaction with devices is becoming ubiquitous: By the end of 2024, it is forecasted that people will be able to interact with over 8.4 billion devices using their voice—larger than the world's population and double the number of devices for 2020 (Moar & Escherich, 2020). At a societal level, inequality in access to technology risks increasing the “digital divide”: worsening existing divisions and increased social exclusion (United Nations, 2016).

Project Relate (“Relate”) is a currently free app from Google available in Android only and can be downloaded from the Google Play Store. Relate enables a person with “nonstandard” speech to create a personalized speech recognition model (in English only) that may better recognize their own speech (Cattiau, 2021).

Relate is argued to reduce the trade-off between AAC speed and accuracy for plwALS as their speech declines, frequently observed when using other forms of AAC (Fried-Oken et al., 2023). Relate is argued to do this by enabling the user to keep talking (maintaining speed) and be better understood by others due to the accuracy of the personalized speech recognition model (maintaining accuracy; Cattiau, 2021).

**Figure 1.**
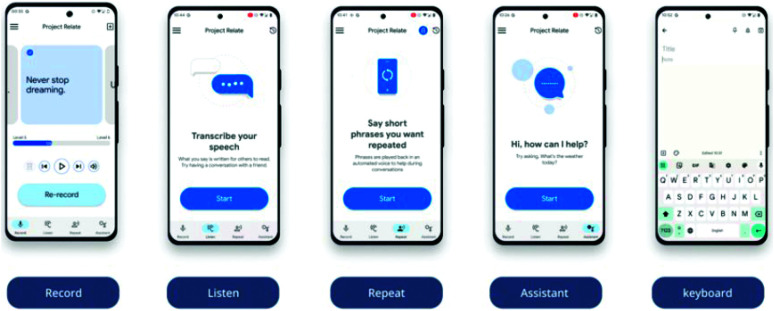
The Relate functions. Printed with permission of Google Research.

Relate has four main features: “Listen,” “Repeat,” “Assistant,” and “Keyboard” (see Figure 1).

A person needs to record a minimum of 500 phrases in Relate in order to create a personalized model (there are more than 5,500 preset phrases available to choose from in the app). Through the “Listen” feature, Relate attempts to transcribe a person's dysarthric speech to text in real time, displaying the text on the phone screen. This is argued to provide the listener with an additional modality to support understanding. However, this feature does not distinguish between speakers and will attempt to transcribe everything the microphone picks up. “Repeat” restates a spoken short phrase of up to 10 s in duration using a synthesized voice. This speech-to-speech functionality is argued to support transactional speech-based conversation without the listener needing to reference text. “Assistant” is argued to enable more successful interaction with Google Assistant using the transcribed speech from Relate. The “Keyboard” function is argued to support a person with nonstandard speech to use the speech-to-text functionality in any app that uses the Google Gboard, for example, Google Docs or Gmail.

Relate additionally has the concept of “custom cards,” which is a way for a person to record a virtually unlimited number of proper nouns or personal phrases that they say in daily life, such as names of family and friends, places they visit, and common phrases (Google, 2021). A person can specify what word or phrase they would like train the ASR model to better recognize and then record it in Relate. Custom cards are argued to support improved personalized ASR accuracy as many of these words may not be present in the general language model that Relate references (Google, 2021). The Relate guidelines encourage recording custom cards as an optional extra activity, and they require at least 24 h to update the model after recording (Google, 2021). As there is no immediate improvement to ASR accuracy when a person records a custom card, planning ahead is important when considering what words to train. To date, there is no identified research on how plwALS use Relate to support everyday spoken conversation with significant communication partners and how any decline in speech intelligibility may affect the usefulness of Relate.

This study focuses on the following objectives: how plwALS and significant communication partners use Relate to support everyday spoken conversation over a period of up to 12 months and how the use of Relate may change with any decline in speech over time. Ethical approval for the project was granted by the University College London Ethics Committee. The outcomes include recommendations for provision of information and training about utilizing ASR technology such as Relate to plwALS, conversation partners, speech-language pathologists (SLPs), and other professionals.

## Method

### Criteria for Participant Recruitment

Three plwALS participants were recruited via an open advert distributed at MND Association branches in the United Kingdom. Each person with ALS nominated a communication partner with whom they communicate on a regular basis. The plwALS were already aware of Relate and were either in the process of recording phrases to create a personalized ASR model or using it already.

All potential participants and their nominated significant communication partners were met by the researcher prior to the formal consent process in person. This enabled potential participants to be provided with information in a clear and understandable form before they decide to progress.

Participants who were included met the following inclusion criteria:

- Participants had a formal diagnosis of ALS or were a communication partner of a person with ALS.
- The plwALS used speech predominantly and already use Relate to some degree.
- The communication partner reports difficulty understanding the natural speech of plwALS without AAC support. The researcher will have ascertained the level of conversational breakdown and repair between plwALS and conversation partners at the interview.
- Participants were over 18 years of age and residents in England, Wales, or Northern Ireland.
- Participants with English as an additional language were accepted if they were able to understand English to a sufficient level as part of the interview process, as judged by the researcher at the information briefing prior to consent.
- There were no restrictions in terms of physical disability, sexual orientation, religion, or ethnicity.

Up to 15% of people diagnosed with ALS develop frontotemporal dementia (FTD), either at the same time or after diagnosis of ALS, and up to 15% of people diagnosed with FTD may go on to develop ALS (Bäumer et al., 2014). People with ALS and FTD may lack the mental capacity to consent to the research or participate throughout the research. Mental capacity is assumed unless there is evidence to think otherwise, in compliance with the Mental Capacity Act 2014 (Office of the Public Guardian, Ministry of Justice, 2014). The researcher is a highly specialized speech and language therapist experienced in completing mental capacity assessments and in working with people with complex communication disabilities. Where there is a concern about capacity to consent or a concern about a participant lacking capacity during the course of the research (who initially had capacity), the participant will be excluded from the research in a sensitive manner.

### Equipment

Between six and 10 conversations were video-recorded and transcribed between each person with ALS and their conversation partner. All recordings were in a quiet room, at either the home of the plwALS or place of work. The recordings were between 10 and 30 min in length.

Video recording equipment was set up by the researcher in the room beforehand. The equipment used to video was a Samsung Galaxy S21 Ultra smartphone, mounted on a stand, and standard Android operating system screen recording software on the smartphone each dyad used to generate the Relate transcriptions from. After each session, the two recordings (video and screen recorder) were merged using Microsoft Clipchamp software, to enable the participants and the Relate captions to be clearly observed on one screen.

The participants were reminded that video recording will take place at the beginning of the session and they were free to stop at any time. The researcher was not in the room to reduce to a minimum any researcher influence on the session and make the recording activity as unobtrusive as possible. The participants were free to talk about anything they wished. There was a possibility that, during video recording, the research participants may reference special category data as defined by the General Data Protection Regulation (U.K. Government, 2018). The research is not collecting these data, and any such reference was audibly masked as soon as possible after the recording has been completed. All information that might lead to identification of the people living with MND (plwMND) or their significant communication partner was audibly masked, anonymized in the written transcripts, and anonymized in the analyses and in any future publications. To better understand how Relate was used by plwALS in their lived experience, there was no training administered between sessions unless help was specifically asked for by the plwALS.

### Assessment of Dysarthria

The Frenchay Dysarthria Assessment–Second Edition (FDA-2; Enderby & Palmer, 2008) was administered to the plwALS after recording after Sessions 1, 3, 6, and 9, as applicable to the number of sessions they participated in. The FDA-2 assessments were administered and scored by the researcher. The intelligibility score for sentences is reported here as it is most relevant to the focus of the research and used to explore changes in conversational speech and change in use of Relate over time.

### Data Analysis

Pseudonyms were used for all participants. All information that might lead to identification of participants was anonymized in transcripts. During analysis, visual details of participants were not masked. For each session, the merged recordings were transcribed using Jeffersonian conventions (Jefferson, 2004). Distributed Open Transcription Environment transcriptions software was utilized (McIlvenny & Davidsen, 2022). Additional records were made for each session, including the number of recordings the plwALS had completed to train Relate and any environmental, technical, or situational factors that could affect Relate caption accuracy.

The number of “custom cards” recorded by plwALS was also noted. A person can record as many custom cards as they wish to try and improve the recognition accuracy of specific nouns and phrases that they use in daily life.

When a personalized ASR model is created or updated, Relate provides the user with an expectation of “high,” “medium,” or “low” ASR accuracy. To do this, Relate calculates a guideline WER score based on a sample of generic phrases tested against the updated ASR model. A “high” rating implies the guideline WER is under 15% (i.e., 15 caption inaccuracies per 100 words spoken), “medium” is between 15% and 35%, and “low” is anything over 35%. The generic phrases are unlikely to be reflective of what a person actually says while using Relate. The researcher additionally calculated WER for the words actually spoken in each recorded session. Relate will attempt to transcribe every speaker it hears and without distinction between the speakers; therefore, WER was only calculated for the speech of the plwALS in the recording. Phrases where speakers overlapped were also disregarded as Relate could not be expected to reliably transcribe only the speech of the plwALS. Phrases were also disregarded where the researcher could not accurately transcribe words spoken after extended analysis. Each video was transcribed as accurately as possible by the researcher: The videos were reviewed multiple times, speech and lip movements were observed, and the context of the conversation was utilized. Occasionally, the plwALS and communication partner were asked to confirm a word or phrase, particularly for proper nouns that the SLP may not have been familiar with.

### Data Analysis: Ecological Validity

Although WER is a commonly used metric for evaluating speech recognition performance (Mishra et al., 2011), it does not consider whether some words may be more important to the meaning of the message and the impact of word errors may be also dependent upon the specific application in which ASR is being used (Favre et al., 2013). Therefore, the researcher additionally allocated a rating for accuracy of the Relate transcription compared to the perceived meaning of what the plwALS actually said, similar to the approach used by Tomanek et al. (2023). This is different to a WER score because it assesses the degree that the underlying meaning was retained even in the presence of ASR caption errors. Transcription meaning accuracy ratings were undertaken by the researcher (a specialist speech therapist with 15 years of experience working with plwALS) and separately by another specialist speech therapist working with plwALS not connected to this research. Any differences of score were discussed, and a rating was agreed. Where a score of 1 or 2 was given, the main cause of the inaccuracy was detailed: incorrect or misspelled proper noun, deletion, insertion, contraction, homophone, repetition, or word error (generally incorrect transcription). Examples for each score are taken from Pam and Carol's first recording. Table 1 shows the rating criteria with example phrases that were scored.

**Table 1.** Example scores for accuracy of transcription.

Score	Description	PlwALS talk	Relate transcription	Main error type
0	Word-for-word transcription accuracy	“It was all good.”	It was all good.	
1	Some transcription inaccuracies; still conveyed enough meaning to reflect what was said	“I said you can talk it's allowed for you to talk she said I can't 'cos the noisy children.”	I said, you can talk it's allowed for you to talk. She said I can't because the noise is children.	Word error
2	Serious transcription inaccuracies; does not convey the meaning of what was said	“And that's for Maxine 'cos they Jim's moving there.”	I like that because it's a movie day and a house. And where was he?	Word error

*Note.* PlwALS = people living with amyotrophic lateral sclerosis.

A meaningfully inaccurate Relate caption may not imply poor understanding of a conversation. For example, the Relate captions may have no effect on understandability if neither party looks at the captions during some or all of talk or relies on another modality to support their understanding. Unlike other forms of AAC (such as a VOCA, for example), Relate does not replace the speech of plwALS, and the interaction does not need to go via the AAC. The ASR captions are generated automatically as a person speaks, and there is no requirement for the listener to utilize or even look at the captions in conversation. This is one reason why it is difficult to link the level of ASR accuracy directly to the level of conversational understanding. The researcher therefore reviewed each videoed session to measure how many seconds either the conversation partner or person with ALS looked at the ASR captions, to get an indication of how frequently they were referenced and what was being captioned when they did. It should be added that a meaningfully accurate Relate transcription may not imply good conversational understanding—this requires comprehension of the meaning, grammar, or phonetic signal of the turn itself as well as perception of how the turn is constructed in relation to previous talk (Bloch & Wilkinson, 2004).

Finally, interviews were undertaken with plwALS and communication partners to explore their feedback on using Relate in conversations. Thematic analysis (TA; Braun & Clarke, 2006) was used for qualitative analysis. The interviews took place as soon as feasible of each recorded interaction using Relate at the preferred location of the plwALS and were conducted by the researcher. A semistructured interview approach was used. This allowed for the flexibility of covering key areas the researcher had planned for and enabled participants to discuss issues important to them that the researcher had not anticipated (Clarke & Braun, 2013). An interview question schedule was created around a set of general questions corresponding to the overall aims of the study and is in the Appendix of this report.

### Participant Details

Pseudonyms were used for all participants. The details of the three dyads in this research are below in Table 2. All three plwALS had intact fine motor skills throughout the study. All three plwALS chose to use the “Listen” function in Relate when in conversation with their communication partners.

**Table 2.** Participants.

Name	Relation	Months since diagnosis	Gender	Role	Primary communication method	AAC	Age (years)
Pam	PlwMND	18	F	Retired	Speech	Pen and paper	60–70
Carol	Friend		F	Retired	Speech	N/A	60–70
Bob	PlwMND	15	M	Working	Speech	Text app on phone	40–50
Adrian	Friend		M	Working	Speech	N/A	30–40
Elsbeth	PlwMND	8	F	Student	Speech	Pen and paper	50–60
Tony	Partner		M	Retired	Speech	N/A	60–70

*Note.* AAC = augmentative and alternative communication; PlwMND = people living with motor neurone disease; F = female; N/A = not applicable; M = male.

Pam had a diagnosis of ALS for about 18 months prior to the beginning of this study. At the time of data collection, Pam was physically well and leading an active lifestyle. Pam presented with moderate mixed spastic–flaccid dysarthria characterized by a harsh voice quality and mild hypernasality. The emergence of moderate articulatory weakness was also noted with moderately reduced tongue movement. Her FDA-2 assessment conversation intelligibility subsection was rated at Grade “C”: “Speech severely distorted; can be understood half the time. Very often has to repeat.”

Carol, Pam's communication partner, has known Pam for more than 40 years. Carol reported that she finds Pam's speech more difficult to understand in recent months and increasingly relies on Pam to write down key words using her whiteboard and pen.

Bob had a diagnosis of ALS for about 15 months prior to the commencement of this study. Throughout the period of research, he was working as an information technology team leader and physically well.

Bob initially presented with moderate mixed spastic–flaccid dysarthria characterized by mild hypernasality; moderate articulatory weakness was also noted with moderately reduced tongue movement. After Session 6, Bob presented with severe spastic–flaccid dysarthria, moderate hypernasality, severe articulatory weakness, and reduced tongue movement. Bob's FDA-2 conversation intelligibility subsection was rated as Grade “C” (“Speech severely distorted—can be understood half the time, very often has to repeat”) after Session 1. After Session 6, Bob's speech was graded as “D” (“Occasional words decipherable”) for the remainder of the study.

Adrian is Bob's friend and work colleague and has known Bob for more than 5 years. Adrian reported that he finds Bob harder to understand over the past year, although they normally still rely on speech to communicate, though occasionally Bob uses his smartphone to type the word he is saying.

Elsbeth is a student and was diagnosed with ALS for 8 months from the start of this research. Her mobility is increasingly impaired.

Elsbeth presented with moderate flaccid dysarthria characterized by low-powered breathy voice quality, moderate articulatory weakness, and moderately reduced tongue movement. Elsbeth's conversation intelligibility rating on the FDA-2 remained at Grade “C” throughout the period of the study (“Speech severely distorted—can be understood half the time, very often has to repeat”).

Tony is her partner of more than 30 years. He reports that he sometimes cannot understand Elsbeth when she initiates a new topic and it helps when she handwrites key words.

Pam reported that she is not confident with technology and relies on her husband for support. She began recording custom cards from Session 5. Bob reported a high level of confidence when using technology and said he had no difficulty with the process of recording phrases and custom cards and using Relate in practice. Bob recorded some custom cards prior to Session 1 and continued through all sessions. Elsbeth reported feeling reasonably comfortable with technology. Similar to Bob, Elsbeth recorded some custom cards prior to Session 1 and through all sessions. All plwALS had been using Relate for a few months prior to the study. Table 2 shows the dyads who participated in the research.

## Results

Although the goal was for approximately monthly video recordings, in practice, this was limited by the availability of the participants over the project's 12-month timeline.

### Dyad 1: Pam and Carol

Pam recorded 3,707 phrases and 70 unique words on custom cards over the period and the Relate app categorized the accuracy of Pam's personalized ASR model recognition as “medium” (average WER between 15% and 35%) throughout. The researcher calculated the actual average WER per session by transcribing the words Pam said and comparing to the ASR captions. Over the sessions, the actual average WER per session varied between 38.2% and 53.2%.

The percentage of Relate captions judged to have preserved the meaning of what Pam actually said varied between the sessions from 26.5% to 49.1%. Further analysis showed that accurate ASR transcription of proper nouns seemed to be a particular difficulty, ranging between 44% and 83% meaning lost for all proper nouns spoken across the sessions. This is significant because proper nouns may not be easily predictable in the sequential context of an interaction to the listener, possibly needing strategies that take longer to repair than other word classes (Bloch & Saldert, 2020).

After Session 5, Pam recorded custom cards to try and improve the ASR caption accuracy of 50 proper nouns, including some names of family and friends. In the subsequent sessions, average meaning loss reduced to 24% for the utterances containing a noun that Pam had recorded custom cards for. Prior to recording custom cards, average meaning loss for utterances containing the specific proper nouns was 70%. This was calculated by assessing all phrases where any of the 50 proper nouns were spoken by Pam and comparing meaning loss before and after custom cards were recorded (see Table 3).

**Table 3.** Pam's forecast and actual WER, meaning preservation percentage, and screen views.

Pam session	FDA-2	Actual average WER	Relate forecast WER	Meaning preserved %	Pam view screen (s)	Pam view screen (% of session)	Carol view screen (s)	Carol view screen (% of session)
Session 1	C	53.2	15%–35%	26.5	2	0.3	70	9.4
Session 2		38.2	15%–35%	45.1	26	2.5	89	8.4
Session 3	C	41.3	15%–35%	49.1	10	1.3	77	9.9
Session 4		48.8	15%–35%	34.0	4	0.5	105	13.0
Session 5		45.5	15%–35%	45.7	6	0.6	53	5.3
Session 6	C	41.8	15%–35%	48.6	0	0.0	50	3.9
Session 7		43.2	15%–35%	43.7	3	0.8	22	6.1
Session 8		44.0	15%–35%	38.3	7	0.9	43	5.4
Session 9	C	46.8	15%–35%	47.0	9	0.9	78	7.5
Session 10		42.1	15%–35%	47.4	4	0.8	25	4.9

*Note.* “FDA-2” Grade C is defined as “speech severely distorted; can be understood half the time. Very often has to repeat.” “Actual average WER” is the WER calculated by phrase for the words actually spoken by the plwALS in each session. “Relate forecast WER” is a guideline WER range that is forecast by the Relate app itself to set user expectations on caption accuracy. “Meaning preserved %” is the average rating for meaning preservation for each phrase spoken by the plwALS in the session. The Relate transcription was compared to the perceived meaning of what the plwALS actually said and assessed for accuracy of preserved meaning by two speech-language pathologists. WER = word error rate; FDA-2 = Frenchay Dysarthria Assessment–Second Edition; plwALS = people living with amyotrophic lateral sclerosis.

Carol and Pam frequently did not look at the ASR captions during conversation. On average, across the sessions, Carol looked at the ASR captions screen for about 7% of the time (average of 60 s in total) for a session lasting, on average, a little over 14 min. Pam looked at Relate even less: about 1% of the time (average of 8 s in total). This is one reason why it is difficult to link the level of ASR caption accuracy directly to the level of conversational understanding. The ASR captions do not replace Pam's speech, and the conversation does not have to go via Relate, unlike some other forms of AAC, such as eye-gaze devices, for example.

Pam reported difficulty with the process of recording phrases and custom cards and frequently enlisted her husband's help. She said the experience of recording was challenging due to fatigue from ALS and her lack of confidence in using technology. Pam more than once blamed herself for the inaccurate captions Relate produced, attributing fault to her own speech and lack of confidence with technology.

Pam reported that she uses Relate only with a few close friends or family and only in certain circumstances—for example, in a quiet environment. She chose to rely on lower technology AAC such as a whiteboard and pen when it was important that her words were exactly understood—for example, when speaking with the doctor. She felt more in control when writing her words rather than relying on automatic captioning.

### Dyad 2: Bob and Adrian

Bob recorded 2,284 phrases and 136 unique words on custom cards over a period of eight sessions (approximately 10 months). Similar to Pam, the Relate app categorized Bob's personalized ASR model recognition accuracy as “medium” throughout the study (average WER between 15% and 35%). The actual average WER per session was calculated and varied between 33.6% and 42.5%. For meaning preservation, the percentage of Relate captions judged to have preserved the meaning of what Bob said varied between the sessions from 27.3% to 50%.

Bob recorded custom cards to try and improve the ASR caption accuracy of 136 proper nouns progressively from Session 2. Bob completed the custom card recordings independently without support. Most of the custom cards contained names of family, friends, people, and places for social activities. There were also some technical terms used in the course of his profession. Bob chose to be recorded at his place of work, and he did not often talk about his family or friends outside of work in the sessions. Across the sessions, Bob said 13 utterances containing one or more words that had already been recorded with a custom card, with an overall proper noun meaning loss of 15% (11 correct, two incorrect). For the 26 utterances with custom cards Bob said prior to the custom card recording, the overall proper noun meaning loss was 69% (eight correct, 18 incorrect). This highlights how guidance may be useful to encourage custom card creation for the contexts where conversation using Relate is likely to occur.

Bob said he felt his speech was declining, and the FDA-2 score after Session 6 confirmed this. Bob appeared to increasingly use Relate to check how accurate the captions were for his speech, perhaps in an attempt to measure his speech decline. Bob did this even when Adrian appeared to understand what he was saying. Bob looked at the captions, on average, 9% of the time from Sessions 1 to 4, increasingly so from Sessions 5 to 8 to around 29% (see Table 4).

**Table 4.** Bob's forecast and actual WER, meaning preservation percentage, and screen views.

Bob session	FDA-2	Actual average WER	Relate forecast WER	Meaning preserved %	Bob view screen (s)	Bob view screen (% of session)	Adrian view screen (s)	Adrian view screen (% of session)
Session 1	C	33.6	15%–35%	50.0	62	10.6	57	9.8
Session 2		39.4	15%–35%	44.0	22	4.5	3	0.6
Session 3	C	41.2	15%–35%	28.9	24	5.0	4	0.8
Session 4		38.9	15%–35%	49.7	90	16.9	25	4.7
Session 5		40.0	15%–35%	29.6	193	29.7	85	13.1
Session 6	D	41.1	15%–35%	30.6	200	28.4	78	11.1
Session 7		40.8	15%–35%	31.4	175	29.3	89	14.9
Session 8		42.5	15%–35%	27.3	210	29.2	85	11.8

*Note.* “FDA-2” Grade C is defined as “speech severely distorted; can be understood half the time. Very often has to repeat.” Grade D is defined as “occasional words decipherable.“ “Actual average WER” is the WER calculated by phrase for the words actually spoken by the plwALS in each session. “Relate forecast WER” is a guideline WER range that is forecast by the Relate app itself to set user expectations on caption accuracy. “Meaning preserved %” is the average rating for meaning preservation for each phrase spoken by the plwALS in the session. The Relate transcription was compared to the perceived meaning of what the plwALS actually said and assessed for accuracy of preserved meaning by two speech-language pathologists. WER = word error rate; FDA-2 = Frenchay Dysarthria Assessment–Second Edition; plwALS = people living with amyotrophic lateral sclerosis.

Bob reported no difficulty with the process of recording phrases and custom cards and using Relate in practice. Bob said that he used Relate occasionally with friends and work colleagues and, similar to Pam, uses other AAC types (texting on his smartphone) when he feels his words need to be precisely understood. Although he voiced concerns about ASR accuracy, he nevertheless felt positive about the potential for ASR technology and what it could do for him. He said Relate should be integrated into the online conference captioning his company uses.

### Dyad 3: Elsbeth and Tony

Elsbeth recorded 1,180 phrases and 120 unique words on custom cards over a period of six sessions. The Relate app categorized Elsbeth's personalized ASR model recognition accuracy as “high” (average WER less than 15%). The actual average WER per session was calculated and varied between 30.6% and 34%. For meaning preservation, the percentage of Relate captions judged to have preserved the meaning of what Elsbeth said varied from 13% to 40%.

Elsbeth recorded 50 custom cards as part of the initial 500 phrases and recorded 70 more over the following 6 months. For the initial 50 custom cards, Elsbeth recorded words predicting what she would likely talk about to her university supervisor—these were all highly technical terms and relevant to her subject area. In the end, she chose not to use Relate in supervision, and almost none of these highly technical words were spoken in the recorded sessions either. For other custom cards, she recorded words were based on specific topics discussed in sessions (such as a trip to the Australian outback—words included “Cassowary” and “Crocodile”), and they were almost never said in subsequent sessions, highlighting the difficulty of predicting what is going to be said in future conversations.

Across the sessions, Elsbeth said 11 utterances containing one or more words that had already been recorded with a custom card, with an overall proper noun meaning loss of 45% (six correct, five incorrect). For the 44 utterances with custom cards spoken by Elsbeth prior to the custom card recording, the overall proper noun meaning loss was 95% (two correct, 42 incorrect; see Table 5).

**Table 5.** Elsbeth forecast and actual WER, meaning preservation percentage, and screen views.

Elsbeth session	FDA-2	Actual average WER	Relate forecast WER	Meaning preserved (%)	Elsbeth view screen (s)	Elsbeth view screen (% of session)	Tony view screen (s)	Tony view screen (% of session)
Session 1	C	31.2	< 15	37.2	2	0.2	225	27.1
Session 2		30.6	< 15	40.0	93	25.5	200	54.8
Session 3	C	33.0	< 15	17.8	25	9.1	121	44.0
Session 4		34.5	< 15	13.0	90	14.6	265	42.9
Session 5		32.0	< 15	18.8	84	9.8	353	41.4
Session 6	C	34.0	< 15	22.4	79	8.9	372	41.8

*Note.* “FDA-2” Grade C is defined as “speech severely distorted; can be understood half the time. Very often has to repeat.” “Actual average WER” is the WER calculated by phrase for the words actually spoken by the plwALS in each session. “Relate forecast WER” is a guideline WER range that is forecast by the Relate app itself to set user expectations on caption accuracy. “Meaning preserved %” is the average rating for meaning preservation for each phrase spoken by the plwALS in the session. The Relate transcription was compared to the perceived meaning of what the plwALS actually said and assessed for accuracy of preserved meaning by two speech-language pathologists. WER = word error rate; FDA-2 = Frenchay Dysarthria Assessment–Second Edition; plwALS = people living with amyotrophic lateral sclerosis.

The ASR captions were mostly referenced by Tony, on average, 40% of the time per session. Elsbeth referenced the captions, on average, 9% of the time. Tony appeared to be checking the accuracy of the ASR captions as he seemed to understand what Elsbeth was saying for much of the time.

Elsbeth reported no difficulty with the process of recording phrases and custom cards. However, the experience of recording was challenging due to fatigue from ALS affecting her voice, and she had to take regular breaks. Elsbeth reported using Relate occasionally with her friends and family but said that “it's not for serious conversations” due to the variable accuracy of the captioning. She wanted to use it when at the university in supervision but decided against trying it because she was concerned how her supervisor may react to any captioning errors. Elsbeth says that talking is part of her sense of identity—she has always liked to talk to people and is hopeful that Relate will become more useful to her as it improves. She felt Relate is the “right concept” to support the way she likes to communicate in life.

## Thematic Analysis (TA)

In TA, a theme should capture something important about the data in relation to the research question, representing some level of patterned response or meaning within the data set (Braun & Clarke, 2006). Following initial coding, five themes were identified: speech and identity, strategies for communication, hopes for personalized ASR, setup and first use, and overall experiences of using ASR in practice.

All three plwMND felt the way they talk and what they say was linked to a sense of who they are and their different roles in life. To be able to keep talking and be understood by friends, family, and work colleagues as speech declines was seen as very important and a way of maintaining important social and work networks. Communication partners supported plwALS trying Relate, believing it may help maintain the confidence of plwMND to participate in activities and maintain networks.

For some, the application was not easy to understand and there was little support available beyond a short training video and family help. Some SLPs appeared to be aware of the app but were reported to having not had the skills to help with practical support. The effort required to record 500 phrases in the face of frequent fatigue (a common symptom of ALS) without any indication of the likely usefulness in daily conversation was seen as a barrier. None found the process of recording custom cards intuitive, despite appearing to be the best way for Relate to better recognize proper nouns such as the names of people, places, and things.

Because of the high perceived level of caption inaccuracy reported by all participants, plwALS indicated they may only use Relate in specific contexts and with particular people, if at all. Communication partners voiced concern at looking away from the plwMND in mid-conversation to the ASR captions—risking a perception that they were not listening, though this view was not shared by plwMND themselves.

For all participants, the hoped-for benefits of personalized ASR were not realized. The captions were seen as too inaccurate in practice, and in the end, some plwMND and conversation partners preferred to resolve any misunderstandings between themselves and not use the ASR captions. One person with MND seemed to attribute responsibility for the ASR captioning errors to her own declining speech, without consideration of the technology shortcomings, risking potentially misplaced negative impacts on their own sense of personal identity.

### Theme 1: Speech and Identity

**“ALS has affected my voice. It hasn't affected my body. But it has affected everything else”** (Pam). For both Pam and Elsbeth, expressing themselves through talk is an integral part of their sense of identity. **“I always talked a lot it's who I am. I'm a talker I can talk for England but I'm hard to understand now”** (Elsbeth). For Bob, voice change and being less easily understood have led to reduced roles in life:** “I used to lead the training but I have handed that over now.”** Communication partners also recognized the effects of voice change:** “The less well you know someone the more embarrassed you can be, but we're past that stage now” **(Carol), and **“I see some others find Bob harder to understand and they talk to him less” **(Adrian).

### Theme 2: Strategies for Communication

Prior to Relate, all were using AAC to support communication when others could not understand what they said: **“I type the word on my phone if someone doesn't understand what I say”** (Bob), **“I write the words down when someone doesn't understand me”** (Elsbeth), and** “I use the whiteboard if I have to be understood. Paul says write the context word first on the whiteboard so I do that” **(Pam). Pam plans ahead when she needs to ask a question: **“I have a note to show people for example can you tell me where the ladies long socks are.”** Alternatively, they get the help of the communication partner to “translate”:** “If someone wants to asks me a question they say ‘where’s Carol?' to help translate they don't understand me without her helping” **(Pam).

Sometimes plwMND use strategies to avoid talking altogether: **“When we walk as a group walk ahead so no one has to talk to me”** (Pam), and** “I don't talk to my neighbours much now I don't want that look of sympathy” **(Elsbeth). Some situations are more difficult to avoid using talk. Elsbeth was completing a PhD and negotiating alternative arrangements for presenting to the upgrade panel:** “They won't understand me so I asked to submit written responses in the upgrade but I am still waiting I have not heard back. It is very worrying.”**

When introduced to Relate, all participants were hopeful it would help them to be better understood when talking:** “If they see my words on the screen they will understand me”** (Pam). The idea that technology could help a person be better understood as they talk was appealing: **“The app is amazing I want to talk just like normal and I want to be understood like normal”** (Bob). Elsbeth is hopeful that it will help her husband: **“You know I told you about this app well it's going to help you understand me better.”** Elsbeth also hoped it would help in her upgrade if the examiners insisted on a verbal interview:** “I hope it won't happen but if I have to talk I will try to use it.”**

### Theme 3: Hopes for Personalized ASR

Communication partners were on the whole positive about the benefits Relate could bring; Tony was concerned that Elsbeth is withdrawing from her social circle: **“She was always arranging things maybe this will help bring her confidence back” **(Tony), and **“This will help Pam—she is so worried about not being understood”** (Carol). Pam was hopeful that people would not finish her sentences for her when using Relate unlike when she was using the whiteboard:** “Relate is quick and no one will wait or finish my sentences like when I'm on the whiteboard.”** However, Tony pointed out that there could be other reasons for not being understood: **“You always tell me it's because I don't listen to you—is this going to make me listen better?”**

### Theme 4: Setup and First Use

Two of the plwMND needed some support to set up Relate and record the phrases in order to create a personalized model:** “I didn't know how to do it I usually leave this sort of thing to Paul” **(Pam). There was not an obvious way to get support: **“My speech therapist didn't know anything about it and there isn't an instruction manual”** (Elsbeth). Pam asked the researcher to visit to help her and her husband better understand how Relate works: “**Thanks I don't think I could have done it without you.”**

Pam felt the number of phrases needed to train Relate was a big commitment:** “Five hundred phrases five hundred it's a lot of work and I get tired.”** Elsbeth felt the same: **“I had to take breaks because my voice got worse as I became tired.” **Bob found recording so many phrases dull:** “I left it for a while as there were more important things to do it was only when you chased me I did anything at all.”**

There was no indication of potential usefulness during the initial recording stage: **“It's boring to do and I don't really know what it will do for me”** (Pam). There was no feedback if a mistake was made, making the task even longer. Pam found out she had made more than a hundred poor-quality or empty recordings before she realized. Relate did not provide any indication on the quality of recordings: **“I thought everything was ok until I listened back to some of the recordings—they were silent or Paul was talking at the same time **…** why can't it tell me when I do it wrong?” **(Pam). The benefit of recording custom cards was not apparent:** “I recorded all the phrases Relate told me to but it didn't say I should record my own words too”** (Pam).

#### First Use of Relate

It was quickly seen by plwMND and communication partners that the ASR captions were frequently inaccurate. However, Carol said Relate sometimes helped her to recognize the correct word even if Relate was not accurate: **“The phonic helps—the word is often wrong but I get the sound of what Pam was saying and that helped me guess.”** Even when few words are transcribed correctly, it could be enough to understand the gist of what has been said:** “A word can give a context—the flow of the sentence is wrong but there are words that are right and there are words that give context and some that sound similar that helps me to jog the meaning in my head”** (Carol).

The plwMND felt Relate was helpful only in specific conversational contexts: **“It is useful for people who don't understand me not with familiar people” **(Bob), and** “I would not use it in serious conversations”** (Elsbeth). Location mattered too: **“I don't used it when I'm out in a crowd with lots of talking”** (Pam),** “It doesn't work well in noisy environments **…** one on one is when I would use it in a quiet room”** (Bob), and **“It was too noisy on holiday to use Relate”** (Pam).

#### Design of Relate

Relate cannot differentiate between speakers and will transcribe every voice it hears as if it is one speaker. This can result in a lot of words on the screen for the reader to review:** “There is a lot of text on the screen and very difficult for me to look at quickly **…** hard to find my way around”** (Adrian).

There were suggestions for how to limit the captions to the plwMND or distinguish who said what words:** “It would help if there is an automatic way of clearing everything quickly from the screen to keep it focused on what is being currently said” **(Adrian). Another idea was color-coding the captions to easily differentiate who said what:** “Can Relate change the colour on the screen for different speakers” **(Adrian).

Bob suggested using a microphone,** “I need a directional microphone so it picks up only my voice not others,” **or to switch Relate on only when he is speaking: **“I prefer to stop and start listen mode so people can read my words and not get confused by other people being transcribed.”** Adrian felt the screen captions were not easy to read:** “I needed to ask to adjust the brightness because I could not see clearly—perhaps this can be done automatically when the app opens,”** and** “The font should be bigger—I can't read the words quickly.” **Carol felt the way the captions roll off the screen made it sometimes difficult to look back to check her understanding as the words were no longer visible:** “I needed to check something earlier to confirm my understanding but it had rolled off the screen and I couldn't see it.”**

Conversation partners were concerned about how plwMND may feel when they look at the Relate screen. Carol believed there would be a negative perception when breaking eye contact to look at Relate:** “Looking away to check the screen feels like it could be rude when you are talking to me.”**

Adrian was aware he was dividing his attention between Bob and the Relate screen:** “In some ways it feelslike you are having a conversation with different people. **…** I want to look at you and not stare at this”** (Adrian). Carol felt it was more “normal” to ask for clarification rather than look at the screen:** “It is just human nature to have the eye contact and say ‘what did you say again?’ **…** Honestly I'm more inclined to say what did you say or go back a bit in a prompt, rather than look because it distracts from conversation because of the relationship of what conversation is.”**

Adrian felt the process of repair was delayed by him looking at Relate and then asking Bob to repeat anyway:** “When I looked a couple of times I became conscious I looked at the screen—I would normally say sorry or ask to repeat something—because I looked at the screen it had been noticed—it feels a bit of a delay to then ask it”** (Adrian). Indeed, Pam took it as a cue to repeat her words when Carol looked at Relate: **“I feel I have got to repeat it again, go back and repeat the sentence”** (Pam).

Overall, plwMND were more comfortable than their conversation partners when either looked at the screen mid-conversation:** “I don't mind if people look at the app but Adrian may not know that”** (Bob),** “I don't mind you looking down at Relate **…** it doesn't feel like a rejection of what I say”** (Pam), and** “I saw him look at the screen but it didn't bother me” **(Elsbeth). Data privacy concerns were raised because Relate attempts to transcribe everyone it hears:** “What I find off-putting is when people around me a proper conversation the words the other person's text to be there which I think like an invasion of privacy for them” **(Bob).

Adrian questioned the need to keep so much transcription history at all: **“You don't need all the history, especially off the screen but it is there. For data privacy concerns it is better to just show the last couple of sentences on the screen and then delete the rest—why have it there at all? It is not likely to be used in the conversation” **(Adrian). Some plwMND sometimes preferred other forms of AAC over Relate as they were more reliable: **“When I use the whiteboard I write the words I want and people don't have any trouble understanding the word **…** I feel I'm in control when I write”** (Pam), and **“I type the right words into my smartphone and show that”** (Bob).

### Theme 5: Overall Experience of Using ASR in Practice

Overall, using the ASR captions in practice was a generally poor experience for both plwMND and communication partners:** “It is accurate at the beginning and then after a while it goes crazy and is not accurate at all—it has a mind of its own”** (Elsbeth). Tony agreed:** “After a while I got a much better idea of what we are talking about directly with Elsbeth”** (Tony),** “It didn't help me at all really today. The words were wrong” **(Pam), and** “Relate was amusing up to a point but some of the words I thought were easy and it should not have got it got them wrong”** (Elsbeth).

Carol felt Relate is not accurate enough:** “Relate does not help in a direct way—not a translation just a hint or clues.”** Adrian agreed:** “Almost every time I looked the word wasn't accurate.”** Tony felt it was not fit for purpose: **“Out of five I score Relate a one.”** Elsbeth summarized her experience: **“It just isn't accurate enough to be used.”** For one person with MND, the negative experience of using Relate extended to a sense of personal failure: **“This is my fault when I'm not understood by Relate” **(Pam).

## Discussion

Relate is argued to reduce the trade-off between AAC speed and accuracy for plwALS as their speech declines, by enabling them keep talking and to be better understood by others due to the accuracy of the personalized speech recognition model (Cattiau, 2021). For many plwALS, there is nothing that could replace the ease, accuracy, and speed of natural speech (Murphy, 2004), and AAC that can increase speed while maintaining accuracy may support maintenance of personal sense of identity, increasing the depth and sophistication of social inclusion, while also maintaining accuracy and authenticity of communication intent (Fried-Oken et al., 2023).

The sense of trade-off from the speed of natural speech to slower or potentially less accurate communication through AAC may feel particularly important to plwMND as they experience progressive communication challenges (Fried-Oken et al., 2023). Removing the need to trade-off between increasing the speed communication while maintaining accuracy may reduce the risks associated with significantly slow rates of communication when using AAC—including social isolation, the risk of negative impact to psychological well-being, and, ultimately, sense of identity (Robillard, 1999).

However, in this study, even though the speed of caption generation approached that of conversational speech, the accuracy of the captions was frequently low, sometimes to the point of distraction, occasionally resulting in slower but more accurate AAC being used instead. Caption accuracy was identified as a significant issue by all participants, although the plwALS felt Relate could be useful in some circumstances.

Actual ASR WER was higher than forecast WER for all plwALS and across almost every session. This may be because all three plwALS used it in conversation, whereas the ASR forecasting process uses a sample of shorter generic phrases. ASR performance is likely to decline for longer utterances or spontaneous conversations (Green et al., 2021). Additionally, the “large language model” that Relate is trained on may have few, if any, examples for some of the proper nouns spoken by the plwALS—for example, the names of people, places, and things the three participants talked about in daily life.

However, the multimodality of communication when using natural speech may be a factor in why we observed less trouble arising from a misunderstanding between plwALS and conversation partners in the videoed interactions than might be expected from the relatively high WER or “meaning lost” scores in isolation. It is difficult to assess for listener understanding based on WER or “meaning lost” scores alone because listeners have more information to understand meaning than only the captions, including the spoken words themselves; tone of voice; pauses; gesture; mood; humor; geographical, social, and educational background; health status; gender; and context—all in addition to the content of the words (Nathanson, 2017). WER and “meaning lost” measures may be closer related to listener understanding if the ASR captions were the only modality available to understand what was being said.

The ASR captions were frequently not referenced by the listener, and as a result, they may have less effect on the interaction than expected by the WER score. It could be argued that caption accuracy matters less when the captions are not being looked at.

We also observed that if an incorrectly transcribed word had similar phonemic and/or semantic properties to the actual word spoken, it was sometimes enough to capture the meaning of what was said or enough to capture context if the ASR captioned a word correctly among incorrectly captioned words. One communication partner said Relate provides “hints and clues” and is not a direct transcription but is still sometimes useful. Another reported that the Relate captions can be helpful when having similar phonemic or semantic properties to the word the plwALS said or if there were some correct words among generally incorrect captioning.

Conversely, the ASR captions sometimes became an additional source of trouble. For example, Bob frequently looked at the captions to the point where he sometimes attempted repair of the incorrect captions even when Adrian showed no indication of trouble understanding. Both Tony and Elsbeth frequently looked at the captions, seemingly to check caption accuracy and which, when they were incorrect, proved to be occasionally a distraction to the conversation thread itself.

Additionally, all three plwALS appeared to prefer spoken word repetition using dysarthric speech when communication partners did not understand them and the Relate captions were incorrect. Pam occasionally used a whiteboard and pen as AAC to write a key word, but seemingly as a last resort. When she did write a key word on the whiteboard, conversation repair was confirmed immediately by Carol. This example illustrates one important difference between ASR captioning and AAC such as a whiteboard. ASR captioning is automatically generated, and any errors it makes are shown on the screen. The ASR cannot “not caption” and generates captions regardless of the level of confidence the internal algorithm has that they are correct. The whiteboard, on the other hand, was completely under the control of Pam to ensure the accuracy of the written word reflected what she meant and could correct it herself immediately if need be and add words as needed.

All three plwALS reported a preference for using speech over AAC, and the ASR captions may be supporting their preference. This is because for the ASR captions to work at all, it requires speech; therefore, the presence of the ASR captions could encourage speech as a modality. Also, the ASR captions do not require either the plwALS or conversation partner to look at the ASR screen at any time—viewing this AAC is not mandatory for it to work—and therefore could be more supportive to plwALS using speech alone than using alternative AAC such as a whiteboard and pen. A whiteboard does not require speech to be used but does require both participants to look at it. In summary, the ASR captions may be perceived as supporting the plwALS preferential use of speech to maintain and/or develop personal relationships, promoting “social closeness” (Murphy, 2004).

Over the period of this study, Bob was the only person with ALS who experienced a decline in speech as measured by the FDA-2. Bob appeared to increasingly use Relate to check how accurate the captions were for his speech, perhaps to measure his speech change. He did this even when his communication partner appeared to understand what was said. Bob progressively looked at the captions more as the sessions progressed, on average 9% of the time from Sessions 1 to 4, increasingly so from Session 5 to 8 to around 29%.

All three plwALS reported that Relate may be only useful in certain contexts and locations and to specific people, if at all. For conversations where it was important to be precisely understood, they all indicated that other forms of AAC were preferred to better ensure the listener understood what they were conveying. All three liked the concept of live captioning of their natural speech and were hopeful that future improved caption accuracy may support them more in everyday communication. The conversation partners all agreed that Relate would be more helpful if it was more accurate. All of them appreciated being able to talk directly to the plwALS while referencing Relate whenever they choose, rather than communicating indirectly via another form of AAC.

Maintenance of personal identity can be impaired by the limitations of AAC devices as well as the disease (Kane et al., 2017), and the limitations of Relate caption accuracy may also have a negative impact on personal identity. One person with ALS blamed her own speech for inaccurate ASR captions, seemingly without taking into consideration any limitations of this relatively new technology itself. Without clarity on both best practices for use and an understanding of the technology limitations of ASR in practical use, there may be an increased risk of misplaced responsibility and ownership of these errors risking negative effects on her own psychological well-being due to ASR performance. Allocating blame to the characteristics of their own “nonstandard” speech rather than the technology for inaccurate speech recognition has been observed in other studies as well (Mengesha et al., 2021).

Finally, the aim of technology such as personalized ASR captions is to support successful conversation (Shor & Emanuel, 2019). However, ASR caption technology is not a replacement for better conversation partner skills, which are worthy of attention in their own right in addition to other communication interventions (Beeke & Bloch, 2023). AAC such as Relate could be viewed as an adjunct to supporting partner skills to meaningful and satisfying conversations (Beeke & Bloch, 2023).

### Limitations

The small size of the sample recruited did not balance across demographic or other characteristics. Further research is needed to determine how representative these findings are across the wider population. As the technology underpinning Relate develops, it is quite possible that the outcomes identified here may change.

### Recommendations

One goal of this research was to inform provision of information and training about Relate to plwMND, significant others, SLPs, and other professionals. The following recommendations are made in the context of the limitations of this research already described.

- Realistic expectation setting: All participants expected Relate to be more accurate than it was. It is important to provide plwALS with accurate information on the expected performance of Relate in the communication scenarios they may wish to use it. PlwALS can then make their own decision whether training Relate is worth the effort or their time.
- Development of a clear and accessible guide to Relate is needed to help meet the individual needs of plwALS, especially those unfamiliar with or lacking confidence in technology.
- A clear and accessible guide should be developed for conversation partners, recognizing that they are also beneficiaries of this technology.
- Relate should be positioned as one of many AAC options available to plwALS, that it is not a “one-size-fits-all solution” and may be better suited in particular contexts, people, or locations and/or used in combination with other AAC types.
- Professionals working with plwALS usually provide information about the progression of the condition and the range of options for communication if and when natural voice deteriorates. This should include an opportunity to discuss personalized ASR technology. The availability of professionals to advise and support people regarding AAC has been shown to improve AAC device uptake (Baxter et al., 2012). Professionals should consider training and using Relate themselves to build their skills and confidence with this tool, helping to build a support network for plwALS who may already be working with them.
- Timing of discussions: The development of guidelines for professionals for how and when to discuss Relate would be helpful. Relate is unlikely to be beneficial when a plwMND is easily understood by others or when speech is already profoundly impaired or lost.
- Specific guidelines for custom cards: It is important that there is awareness raising and training on how to get the best from them.
- The way Relate measures forecast accuracy of the personalized ASR model could be reviewed. In this research, the forecast WER was almost always lower than the actual WER. Additionally, as the technology aims to support listener understanding, then a measure of ASR accuracy could include feedback from the listeners themselves.

## Data Availability Statement

The data sets generated and/or analyzed during the current study are available from the corresponding author on reasonable request.

## Notes

**Publisher Note:** This article is part of the Forum: Research Symposium on Artificial Intelligence.

## Appendix

### Interview Sheet

- Overall how have you been finding using Relate?
- Any particular highlights, positive or negative?
- What have been people's reactions when you used Relate?
- Did you find that the way you used the application changed over time?
- Are there any situations in which you thought Relate could be useful, but not the case anymore?
- Are there any situations in which you thought Relate would not be useful, but was?
- For the conversation partner: How hard did you work to understand what was said? Remember, this is different than how many words you got right. For example, you could get all the words right but have to work very hard to do it (score out of 10, 10 being a lot of work).
- Is there anything else you would like to add?
